# Ce/Mn/Cr: (Re,Y)_3_Al_5_O_12_ Phosphor Ceramics (Re = Gd, Tb and Lu) for White LED Lighting **with** Significant Spectral Redshift and Improved Color-Rendering Index

**DOI:** 10.3390/ma16206667

**Published:** 2023-10-12

**Authors:** Yukun Li, Svenja Hanson, Cheng Heng Pang, Peng Lyu, Jun Jiang

**Affiliations:** 1 Ningbo Institute of Materials Technology and Engineering, Chinese Academy of Sciences, 1219 West Zhongguan Road, Ningbo 315201, China; liyukun@nimte.ac.cn; 2 Department of Chemical and Environment Engineering, University of Nottingham Ningbo China, 199 Taikang East Road, Ningbo 315201, China; chengheng.pang@nottingham.edu.cn; 3 Bradley Department of Electrical and Computer Engineering, Center for Photonics Technology, Virginia Tech, Blacksburg, VA 24061, USA; lpalb@vt.ed

**Keywords:** garnet phosphor, laser lighting, full spectrum, spectral modulation, Ce/Mn/Cr: (Re,Y)_3_Al_5_O_12_

## Abstract

In order to attain phosphor ceramics with a high Color-Rendering Index (CRI), samples with the composition of Y_0.997−x_Re_x_Ce_0.003_)_3_(Al_0.9748_ Mn^2+^_0.024_Cr^3+^_0.0012_)_5_O_12_(Re_x_ = 0, Gd_0.333_, Gd_0.666_, Gd_0.997_, Tb_0.333_, Tb_0.666_, Tb_0.997_ and Lu_0.997_ were prepared by solid-state reaction and vacuum sintering, and exhibited potential for high-quality, solid-state lighting. Doping with Cr^3+^ and Mn^2+^ effectively enhanced the red component of Ce^3+^ spectra through the intense energy transfer from Ce^3+^ ions to Mn^2+^/Cr^3+^ ions. The crystal field splitting of [GdO_8_] and [TbO_8_] was more extensive than that of [YO_8_], causing a massive redshift in the Ce^3+^ emission peaks from 542 to 561 and 595 nm, while [LuO_8_] had an opposite effect and caused a blueshift with a peak position at 512 nm. White LED devices incorporating Ce/Mn/Cr: (Gd_0.333_Y_0.664_)_3_Al_5_O_12_ phosphor ceramic exhibited a high CRI of 83.97, highlighting the potential for enhancing the red-light component of white LED lighting.

## 1. Introduction

White light-emitting diodes (WLEDs) are gaining popularity in solid-state lighting because of their high luminous effectiveness, extended lifespan, small size, environmental friendliness and affordable production method. Ce: YAG transparent ceramics are fast evolving as the next generation of color-converted material without the drawbacks of typical Ce: YAG phosphors, such as their inferior mechanical robustness, poor heat conductivity and dissatisfactory lumen depreciation [1,2,3,4]. Blue InGaN chips are often used in conjunction with Ce: YAG phosphors in current white illumination. This scheme allows the transmission of blue light to mix with the output yellow light to create white light. Nevertheless, this technique will have a low CRI value since Ce: YAG spectra lack red and green components.

Two basic approaches have been taken to address this issue. Ce: YAG may shift its emission spectrum toward longer wavelengths by changing the material’s chemical structure, or the emission spectrum’s red component can be boosted by adding luminescence centers [5,6,7,8,9,10,11]. Cerium ions can exist in either a trivalent state, Ce^3+^, or a tetravalent state, Ce^4+^. Ce^4+^ is non-luminescent, whereas Ce^3+^ ions exhibit luminescence [12]. For the first method, the luminescence performance of Ce^3+^ is impacted by the crystal-field splitting phenomenon when the local crystal field is altered. The crystal field splitting can be enhanced, and the accompanying redshifts of the emission spectra can be realized by increasing the diameter of the ion in the dodecahedral position or decreasing its diameter in the octahedral position in the YAG lattice, respectively. The ion species Tb^3+^, Gd^3+^, Lu^3+^, Mg^3+^, Sc^3+^, etc., are claimed to substitute Y^3+^ at the dodecahedral position in Ce: YAG in the recently described garnet system fluorescent ceramics. Ga^3+^, Mg^3+^, Sc^3+^, etc., may replace Al^3+^ at the location, whereas Si^3+^ can replace Al^3+^ at the tetrahedral site. For example, a new plate ceramic phosphor Ce^3+^: Lu_2_Mg_2_Al_2_Si_2_O_12_ has been fabricated for laser-driven lighting. It emits a broad yellow–orange light with a bandwidth of 130 nm and improved thermal stability compared to other phosphors [13]. Fluorescent ceramics Ce: (Tb,Gd)_3_Al_5_O_12_ with good characteristics were developed by Chen et al. [14], the spitting of the crystal field was further amplified, and the spectra were more redshifted by substituting Gd^3+^ for Tb^3+^ at the dodecahedron sites. The emission peak moves from 550 nm to 570 nm when Gd^3+^ doping rises, and the light output is significantly boosted. Researchers at Henan Polytechnic University [15] used Sparkling Plasma Sintered LuAG: Ce transparent ceramics as a green light emission converter for high-power, laser-driven lighting. The primary emission wavelength is 523 nm when excited by a 445 nm blue light laser, with an external quantum efficiency of 77%.

In terms of the incorporation of the luminescence centers method, many nitride and fluoride red-emitting phosphors have been developed and studied extensively for their high conversion efficiency of blue light, suitable emission color and low thermal quenching. Xie et al. [16] fabricated a complex phosphor consisting of Eu^2+^: Sr_2_Si_5_N_8_ and Eu^2+^: Sr_2_SiO_4_ by solid-state reaction. The resulting phosphor emits an intense orange–red light in the red region, with a wavelength range of 616–670 nm. Brinkley et al. [17] combined Eu^2+^: Sr_2_Si_5_N_8_ with Ce: YAG phosphors to improve white lighting properties via being excited by blue LEDs, and the luminous efficacies reached 94 lm/W with a CRI of 72. The Eu^2+^: Sr_2_Si_5_N_8_ phosphors were also found to own decent thermal stability of the remaining 72% at 150 °C compared to room temperature. Pust et al. [18] obtained narrow-band red light Eu^2+^: Sr[LiAl_3_N_4_] phosphors efficiently excitable by blue LEDs. The peak position of the spectrum is located at 650 nm with an FWHM of about 50 nm, and excellent thermal stability was also demonstrated by maintaining more than 95% quantum efficiency at 200 °C relative to that at RT. High-performance red phosphors Eu^2+^: Sr[Li_2_Al_2_O_2_N_2_] were fabricated by Hoerder et al. [19] displaying excellent optical properties such as an optimal spectral position, small spectral full width at half maximum and exceptional thermal stability. The phosphor-converted LED prototype showed a 16% increase in luminous efficacy compared to high color-rendering phosphor-converted LEDs. Among these nitride red phosphors, Eu^2+^: CaAlSiN_3_ is the most mature and commercialized, and related research has been ongoing in recent years [20,21,22].

Regarding research on fluoride red phosphors, a new, red fluoride aluminate phosphor, Mn^4+^: Na_3_AlF_6_, has been synthesized at room temperature using a two-step method [23]. This phosphor exhibits bright red fluorescence at 630 nm. The optimal composition of 1.58%Mn^4+^: Na_3_AlF_6_ shows excellent thermal stability and is used to create a warm, high-power white LED with a high Color-Rendering Index (Ra = 92.7 and R9 = 94) and low-color temperature (CCT = 3903 K). Another new and efficient red phosphor, BaGeF_6_:Mn^4+^, has been fabricated by a hydrothermally etching method [24]. The phosphor has broad adsorption and sharp emissions in blue and red ranges. Wang et al. [23] combined narrow-band green phosphors Eu^2+^: β-sialon and red phosphors Mn^4+^: K_2_SiF_6_ with a blue InGaN chip to create white light-emitting diodes. The green phosphor has a peak emission at 535 nm, an FWHM of 54 nm, and an external quantum efficiency of 54.0%. The red phosphor, K_2_SiF_6_:Mn^4+^, has a sharp line emission spectrum with the most intense peak at 631 nm, an FWHM of ~3 nm and an external quantum efficiency of 54.5%. However, using nitride phosphors or nitride phosphors in glass (PiG) as light-converted materials still poses a challenge due to the low thermal conductivity of silicone gel or glass matrix, which limits their application in high-power/high-brightness white lighting. While Ce: YAG/nitride composite ceramics are adopted, the disadvantages of nitride ceramics, such as a low diffusion coefficient, high saturation vapor pressure and the easy occurrence of chemical reactions at high temperatures, make achieving dense sintering difficult. Additionally, ceramic composites consisting of Ce: YAG ceramic/fluoride ceramics will lead to a drop in luminous efficacy due to the absorption of green and yellow emissions. Therefore, it is worth exploring the improvement of light quality within a single-phase ceramic phosphor, such as Ce^3+^/Pr^3+^/Cr^3+^: YAG [25], Ce^3+^/Mn^3+^/Si^3+^: YAG [26], Ce^3+^/Mn^2+^/Si^4+^: Y_3_Al_5_O_12_ [27], Ce^3+^/Mn^2+^: Lu_3_Al_5_O_12_ [28], Dy^3+^/Cr^3+^: YAG [29], Cr^3+^: YAG [30], Eu^3+^: Li_6_CaLa_2_Sb_2_O_12_ [31], Eu: Ca-α-SiAlON [32], Eu: Y_2_Mo_4_O_15_ [33] et al. By using the above fluorescent ceramic material system, the fluorescence performance is significantly improved, achieving a redshift of emission spectra, a reduction in correlated color temperature, and an improvement in the Color-Rendering Index across the board.

Given that both altering lattice structures and introducing multi-ion luminescent centers are effective methods for spectral modulation and achieving a high Color-Rendering Index, it is worthwhile to explore the simultaneous utilization of both approaches within a single ceramic structure to achieve spectral redshift and enhance the red portion of the spectrum. Currently, there is little research combining these two methods to achieve a high CRI of phosphor ceramics. Previously, we reported Ce/Mn/Cr: Y_3_Al_5_O_12_ phosphor ceramics [34]. Intense energy transfer from Ce^3+^ to Mn^2+^/Cr^3+^ is obtained by both non-radiative and radiative processes, demonstrating the versatility of Ce^3+^ as activators and sensitizers. Under 450 nm laser diode irradiation, a high CRI value of 75.3 is produced in the sample with the optimal doping concentration. Based on the previous research, we further fabricated Ce/Mn/Cr: (Re,Y)_3_Al_5_O_12_ (Re = Gd, Tb and Lu) phosphor ceramics. Naturally, the energy transfer process from Ce^3+^ to Mn^2+^/Cr^3+^ is similar, but the introduction of Gd, Tb and Lu further modifies the 5d crystal field splitting of Ce^3+^, modulates the spectrum and consequently alters its luminescent performance, and a high CRI of 83.97 was successfully achieved. In-depth studies were conducted on the developed phosphor ceramics’ phase composition, microstructures and photoluminescent capabilities. As the last step, the luminous qualities of the fabricated phosphor ceramics under blue LED stimulation were tested.

## 2. Experimental

The transparent ceramics (abbreviated as TCs) were manufactured using a solid-state reaction and vacuum sintering. Precisely, Gd_2_O_3_ (Aladdin Co., 99.99%), Lu_2_O_3_ (Aladdin Co., 99.99%), Tb_4_O_7_ (Alfa Aesar Co., 99.99%), Y_2_O_3_ (Aladdin Co., 99.99%), Al_2_O_3_ (Aladdin Co., 99.99%), Ce_2_(CO_3_)_3_ (Aladdin Co., 99.99%), Cr_2_O_3_ (Aladdin Co., 99.95%) and MnCO_3_ (Aladdin Co., 99.95%) were weighed out based on the stoichiometric compositions of (Y_0.997−x_Re_x_Ce_0.003_)_3_(Al_0.9748_ Mn^2+^_0.024_Cr^3+^_0.0012_)_5_O_12_(Re_x_ = 0, Gd_0.333_, Gd_0.666_, Gd_0.997_, Tb_0.333_, Tb_0.666_, Tb_0.997_ and Lu_0.997_, respectively). Table 1 lists the quantity and precise chemical makeup of the substances under examination. The initial powders were combined with ZrO_2_ balls in ethyl alcohol for 8 h at 200 revolutions per minute in a ball mill (MITR-YXQM-2L, Mitr, Changsha, China) . The resulting slurry was then dried in an oven (DHG-9070A, Yiheng, Shanghai, China) at 85 °C and pulverized in an agate mortar. Calcination (L40/12/P330, Fischer, Achern, Germany) at 900 °C for 2 h was used to remove organic components from the powders. The pellets were formed by passing the calcined powders through a 200-mesh filter and pressing them mechanically into steel molds (Φ = 15 mm) (769YP-40, Ahbeq, Anhui, China). After cold isostatic pressing at 200 MPa to generate green bodies, the designed TCs are sintered at 1700–1730 °C for 3–6 h in a vacuum environment of 10^−3^ Pa (CXZW-45-20sh, Sh-vac, Shanghai, China). As a final step, the TCs were polished (Labopol-5, Struers, Copenhagen, Denmark)on both sides and optionally annealed in an air atmosphere for further characterization.

X-ray diffraction (D8, Bruker, Karlsruhe Germany)using Cu K radiation with a step size of 0.02° and a scanning range of 10-0° was used to determine the crystalline phase compositions. A UV/VIS/NIR spectrophotometer (Model Lambda 950, Perkin Elmer, US) was used to analyze the in-line transmission spectra. A fluorescence spectrophotometer (F-4600, Hitachi, Chiyoda City, Japan) with a xenon lamp for excitation was used to examine both photoluminescence (PL) and photoluminescence excitation (PLE) spectra. Another fluorescence spectrometer (F-311, Horiba, Osaka, Japan) was used to measure the Ce^3+^ luminescence lifetime. An integrating sphere system (Labsphere, North Sutton, NH, USA) equipped with a multichannel photodetector (MCPD-9800, Otsuka Photal Electronics, Chiyoda City, Japan) was used to take readings of the TCs’ electroluminescence spectra, luminous flux, Correlated Color Temperature (CCT), Commission Internationale de L’Eclairage (CIE) chromaticity coordinates, and Color-Rendering Index (CRI).

## 3. Results and Discussions

**Figure 1:**
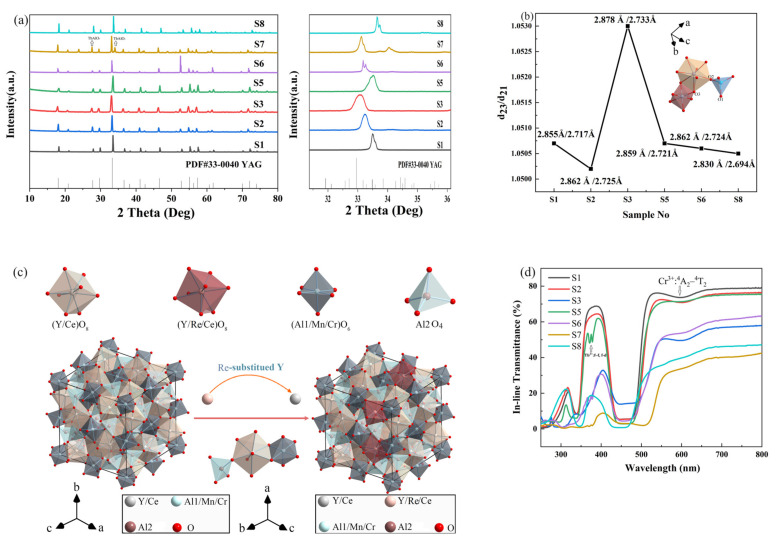
(**a**) Ce/Mn/Cr: (Re,Y)_3_Al_5_O_12_ TCs X-ray diffraction patterns at varying Re-doping Levels (left) and enlarged patterns from 31° to 36° (right). (**b**) The ratio of d_23_/d_21_ for Ce/Mn/Cr: (Re,Y)_3_Al_5_O_12_ TCs calculated from Rietveld refinement methods by GSAS II software; d_23_ is defined as the distance between oxygen atoms O2 and O3; d_21_ is defined as the distance between oxygen atoms O2 and O1. (**c**) Crystal structure schematic of the Ce/Mn/Cr: (Re,Y)_3_Al_5_O_12_ ceramics. The (Y/Re/Ce)O_8_ dodecahedron, the (Al1/Mn/Cr)O_6_ octahedron and the Al_2_O_4_ tetrahedron are exhibited next to and independently from one another. (**d**) Spectra of in-line transmission through 1.0 mm thick Ce/Mn/Cr: (Re,Y)_3_Al_5_O_12_ TCs after double-sided polishing.

Figure 1a shows the XRD patterns of the planned Ce/Mn/Cr: (Re,Y)_3_Al_5_O_12_ TCs. Except for S7, all of the doping Ce^3+^/Mn^2+^/Cr^3+^/Si^4+^ ions are capable of entering the YAG lattice in their entirety since all of the samples are adequately matched with the standard YAG card (PDF#33-0040) and there are no second phases or other contaminants identified. For S4, once Gd^3+^ fully replaces Y^3+^ in the lattice to create GdAG, the resulting material is thermodynamically unstable and breaks down into Al_2_O_3_ and GdAlO_3_ at about 1500 C [35]. In addition, for S7, the TbAlO_3_ second phase is formed when Tb^3+^ completely replaces the Y^3+^ in dodecahedral sites. The optical quality of the ceramic will suffer greatly if the second phase occurs during production. Moreover, as seen in Figure 1a, the diffraction peaks monotonically shift towards a lower angle when the Gd^3+^ or Tb^3+^ doping concentration slowly rises, proving that the lattice expansion occurs by bigger Gd^3+^(1.053 Å) or Tb^3+^ (1.04 Å) substitution of smaller Y^3+^ (1.019) [36,37], as per Bragg’s Law. Conversely, when smaller Lu^3+^ ions (0.977 Å) [38] replace Y^3+^ ions, the lattice shrinks and the XRD curve shifts in the direction of the high angle. In Figure 1b, d23 and d21 represent the O2-O3 and O2-O1 distance, respectively, and the specific positions of the O1, O2 and O3 atoms within the lattice can be observed in the structural schematic diagram shown in figure, while d23/d21 can be used to characterize the lattice symmetry and the extent of crystal field splitting, which will be discussed later. Figure 1c depicts the crystal structure schematic of the Ce/Mn/Cr: (Re,Y)_3_Al_5_O_12_ ceramics, and the positions of each atom and possible substitution modes are also displayed.

**Figure 2:**
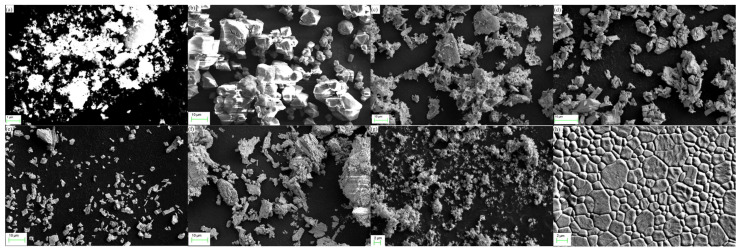
SEM images of Y_2_O_3_ (**a**), Al_2_O_3_ (**b**), Gd_2_O_3_ (**c**), Tb_4_O_7_ (**d**), Lu_2_O_3_ (**e**), Ce_2_(CO_3_)_3_ (**f**) raw material powders, S2 powders after ball milling (**g**), S2 TC surface (**h**).

Figure 1d presents the optical transmittance spectra of S1–S7. As seen in Figure 2, S1, S2 and S5 samples showed good transmittance, where the highest transmission rate is 78.9% at 800 nm, achieved by S1. For S2 and S5, the transmittance also exceeds 75% at 800 nm. As the concentration of Gd^3+^ or Tb^3+^ increases, the transparency of the sample shows a significant decreasing trend. Ce^3+^: 4 f → 5 d^2^ and 4 f → 5 d^1^ transitions are responsible for the two large absorption bands around 340 nm and 440–480 nm, respectively [39]. The Cr^3+^ ion’s ^4^A_2_ → ^4^T_2_ transitions manifest within the absorption bands approximately at 600 nm [40]. In S5 and S6, the absorption band between 371–379 nm was ascribed to the ^7^F_6_ → ^5^D_2_ transitions of Tb^3+^ [14]. There should have been more absorption bands of Tb^3+^ observed in the transmittance spectra, such as bands at 324–335 nm (^7^F_6_ → E_1_ transition), 354 nm (^7^F_6_ → ^5^D_2_ transition) and 483 nm (^7^F_6_ → ^5^D_4_ transition) [41]. Nevertheless, all of them are submerged in the strong absorption band of Ce^3+^ and cannot be seen apparently. It is worth mentioning that in S8 the crystal field splitting of [LuO_8_] is smaller than that of the other samples, resulting in a rise in the ^5^d_1_ energy level and a sinking of the ^5^d_2_ energy level, which in turn causes a redshift in the absorption of Ce^3+^ around 340 nm and a blueshift around 450 nm.

The shapes of the raw materials are illustrated in Figure 2. The Y_2_O_3_, Al_2_O_3_, Gd_2_O_3_, Tb_4_O_7_ (d), Lu_2_O_3_ and Ce_2_(CO_3_)_3_ powders exhibit diverse morphologies, with particle size distributions ranging from 1 to 20 μm. The Y_2_O_3_ powder consists mostly of blocky or plate-like particles with an average particle size of 500 nm. The Al_2_O_3_ powder is blocky and exhibits significant aggregation, with an average particle size of approximately 3–5 μm. Gd_2_O_3_ powder presents a flake-like morphology, with the majority of particles ranging in size from 2–10 μm. Tb_4_O_7_ powder has a relatively uniform particle distribution, with an average grain size of about 3 μm, and Lu_2_O_3_ raw material powder exhibits good dispersion characteristics. Through ball milling, the aggregation of powders is significantly improved with the average particle size around 2 μm. In the case of TC S2, it demonstrates a compact surface microstructure characterized by well-defined boundaries and minimal pores. The predominant grain size falls within the range of 1–5 μm.

**Figure 3:**
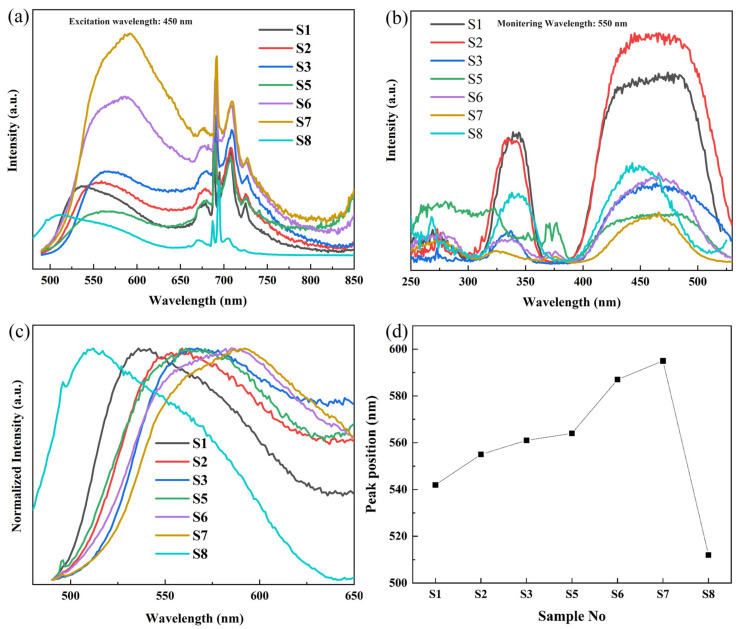
(**a**) PL spectra between 480 and 850 nm from S1 to S8. (**b**) PLE spectra between 250 and 530 nm from S1 to S8. (**c**) Normalized PL spectra between 480 and 650 nm from S1 to S8. (**d**) Peak positions of PL spectra as a function of Re^3+^ concentration.

Figure 3a displays the PL spectra between 480 nm and 850 nm of samples S1, S2, S3, S5, S6, S7 and S8 stimulated by a 450 nm pumping source. All spectra displayed a characteristic yellow emission from 500 to 650 nm, attributed to the ^5^d_1_ → 4 f electron transition of Ce^3+^. Figure 3b illustrates the PLE spectra between 250 and 530 nm with the monitoring wavelength of 550 nm, from S1 to S8. Due to the crystal-field splitting, two excitation bands were formed. The one peaked at 340 nm is due to the 4 f → ^5^d_1_ electron transitions of Ce^3+^, and the other one between 380 and 520 nm derives from the 4 f → ^5^d_1_ electron transitions and corresponds nicely to the mature blue light chips.

The Cr^3+^ was designed to occupy the Al^3+^ position in the octahedron. The lowest excited state is ^2^E level and the major radiative decay comes from the ^2^E → ^4^A_2_ non-phonon transition, generating light with a maximum wavelength of 689 nm. The emission peaks at 707 nm (Stokes sideband) and 725 nm are low-frequency R-line sidebands, while the peak at 677 nm is the anti-Stokes, high-frequency R-line sideband [30]. Meanwhile, Mn^2+^ ions in dodecahedral and octahedral sites can emit light in the orange and red regions [42]. Hence, by introducing the Cr^3+^/Mn^2+^ doping, the red light in the visible light area may be compensated, and this optimization can presumably benefit the acquisition of the whole spectrum and the enhancement of the CRI. Essentially, the energy transfer can be realized from Ce^3+^ → Mn^2+^ and Ce^3+^ → Cr^3+^. The former is a non-radiative process while the latter consists of radiative and non-radiative components. The electron transition and luminescence principle of Cr^3+^ and Mn^2+^ in the YAG crystal lattice, as well as the energy transfer mechanism from Ce^3+^ to Cr^3+^/Mn^2+^, were discussed in our previous study [34]. In this research, we focus on the shift of emission bands in [CeO_8_] caused by substituting Gd^3+^, Tb^3+^and Lu^3+^ for Y^3+^.

Figure 3c shows the normalized PL spectra between 480 and 650 nm from S1 to S8. The emission peak for S1 is 542 nm, and with more Gd^3+^ doping into the YAG lattice to replace Y^3+^, the emission peak redshifted to 555 nm and 561 nm for S2 and S3, respectively. As the concentration of Tb^3+^ increases, the peak wavelengths of S5, S6 and S7 redshift to 564 nm, 587 nm and 595 nm, respectively. In addition, the substitution of Y^3+^ with Gd^3+^ broadens the emission band. The summary of the variation in peak wavelengths for different samples is also presented in Figure 3d. As shown in Figure 3b, for S2 the narrow excitation band around 275 nm, which does not show in the excitation spectra of Ce: YAG, is created by the ^8^S_7/2_ → ^6^I_J_ electron transition of Gd^3+^ [43]. For S5, S6 and S7, the excitation bands between 250 and 324 nm are ascribed to the 4 f-5 d transition of Tb^3+^. The excitation bands peaking at 376 nm corresponded to the Tb^3+^:^7^F_6_-^5^D_3_ transitions and the ones peaking at 483 nm were due to Tb^3+^:^7^F_6_-^5^D_4_ [41]. This excitation band at 483 nm of Tb^3+^ overlaps with the broad main excitation bands of the Ce^3+^, demonstrating that Tb^3+^ possibly acts as a sensitizer for the Ce^3+^ ions [44]. For S8, the spectra exhibited typical broadband emission with a peak positioned at 512 nm. The blueshift in the emission of Ce: LuAG transparent ceramic is due to the weaker crystal field splitting between the 5 d_1_ and 5 d_2_ in the LuAG crystal lattice [15]. As a result, the excitation at 340 nm is redshifted while the excitation at 450 nm is blueshifted due to the weakening of the crystal field cleavage.

Song et al. [45] stated that the [CeO_8_] symmetry distortion strength and the resulting crystal field splitting (CFS) can be determined by the O2-O3 and O2-O1 distance ratio, represented as d_23_ and d_21_, respectively. The higher the d23/d21 ratio value, the more significant the [CeO_8_] distortion and CFS. The d_23_/d_21_ values from S1 to S8 are shown in Figure 1b. It is observed that although the S3 sample exhibits a significantly higher d_23_/d_21_ value and a pronounced redshift in its spectrum, there is no clear correlation between the degree of redshift in the spectra of the other samples and the magnitude of their respective d_23_/d_21_ ratios. Except for the symmetry of the Ce^3+^ position within the crystal lattice, the magnitude of crystal field splitting is also associated with various other factors, such as the bond length between Ce^3+^ and coordinating anions, the degree of overlap of molecular orbitals and the coordination environment [46].

**Figure 4:**
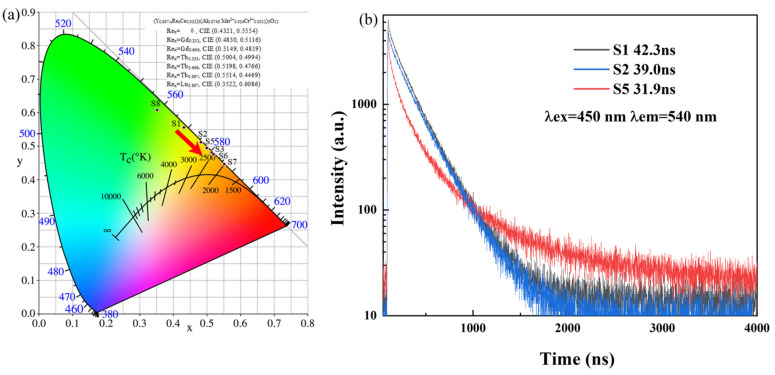
(**a**) CIE chromaticity diagram for the Ce/Mn/Cr: (Re,Y)_3_Al_5_O_12_ TCs; isothermal lines representing the same CCT are included in the diagram. (**b**) Fluorescent decay curves of Ce/Mn/Cr: (Re,Y)_3_Al_5_O_12_ TCs.

As seen in Figure 4a, the transparent ceramics were analyzed from their emission spectra to have the CIE color coordinates of around (0.4321, 0.5554), (0.4830, 0.5116), (0.5149, 0.4819), (0.5004, 0.4994) (0.5198, 0.4766) (0.5514, 0.4469) and (0.3522, 0.6086) for S1, S2, S3, S5, S6, S7 and S8, respectively. Correspondently, the CCT of the designed Ce/Mn/Cr: (Re_x_Y_1−x_)_3_Al_5_O_12_ TCs may be varied from 3922 K to 3081, 2568, 2819, 2489, 2060 and 5198 K by controlling the types and quantity of Re-doping, which demonstrates the enormous potential of the Ce/Mn/Cr: (Re,Y)_3_A1_5_0_12_ TCs for producing warm white light with a high CRI value, since the CCT values are remarkably lower than that of the commercial Ce: YAG phosphor (~6000 K). Due to the subsequent photoluminescence performance testing, we employed the transmission mode for white light illumination of the fluorescent ceramics. Therefore, in this study series, we focus more on samples S1, S2 and S5, which exhibit better transparency.

Figure 4b depicts the decay kinetics of the Ce^3+^ emission for Ce/Mn/Cr: (Re,Y)_3_Al_5_O_12_TCs under 450 nm excitations. The fluorescence lifetimes of S1, S2 and S5 can be obtained by fitting the decay curve using a bi-exponential model described by Equation (1):

*I*(*t*) = *I*_0_ + *A*_1_
*exp* (−*t*∕*τ*_1_) + *A*_2_
*exp* (−*t*∕*τ*_2_) (1)

where *I*(*t*) represents the instantaneous intensity of luminescence at time *t*, *τ*_1_ and *τ*_2_ correspond to the rapid and slow components of the lifetime, respectively, and *I*_0_, *A*_1_ and *A*_2_ are constants. The average lifetime *τ* can be calculated using Equation (2) as follows [6,47]:

*τ* = (*A*_1_*τ*_1_^2^ + *A*_2_*τ*_2_^2^)∕(*A*_1_*τ*_1_ + *A*_2_*τ*_2_) (2)

Using the preceding equations, the fluorescent decay lifetimes of Ce^3+^ were estimated as 42.3, 39.0 and 31.9 ns for S1, S2 and S5, respectively.

**Figure 5:**
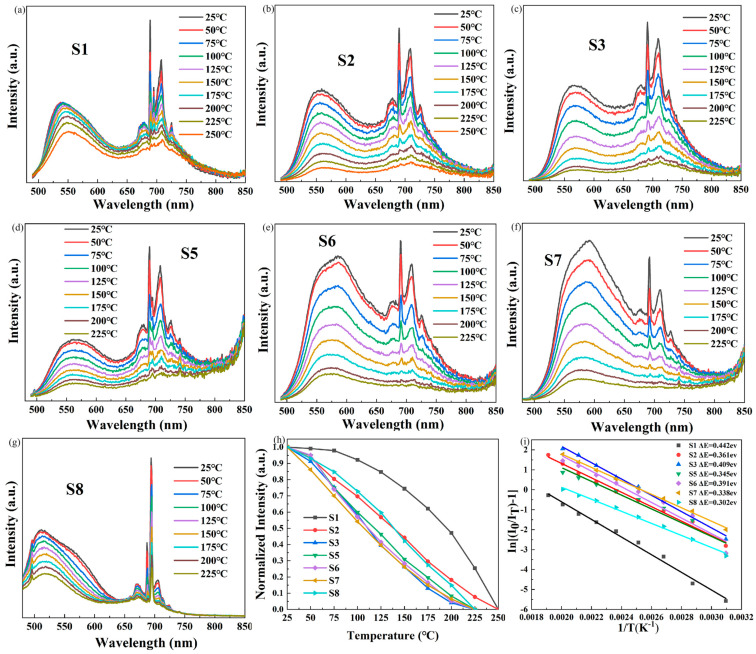
Temperature-dependent PL spectra of the Ce/Mn/Cr: (Re,Y)_3_Al_5_O_12_TCs of S1 (**a**), S2 (**b**), S3 (**c**), S5 (**d**) S6 (**e**), S7 (**f**) and S8 (**g**) under the 450 nm pumping source. (**h**) Normalized peak position intensities of the Ce/Mn/Cr: (Re,Y)_3_Al_5_O_12_TCs with varying temperatures. (**i**) Plots of Ln (I_0_/I_T_-1) versus 1/T of Ce/Mn/Cr: (Re,Y)_3_Al_5_O_12_TCs.

Figure 5 depicts the temperature-dependent PL spectra of Ce/Mn/Cr: (Re,Y)_3_Al_5_O_12_ TCs with varying concentrations of Re^3+^. All samples exhibit a steady decline in PL intensities with increasing temperature, as would be expected. S1 has the best thermal stability, maintaining 92.2% of the fluorescence intensity at 100 °C compared to that at room temperature, followed by the S2 and S8 samples, keeping 70.0% and 72.8% fluorescence intensity at 100 °C of the initial measurement value. In contrast, the thermal stability of the other samples was relatively average.

**Figure 6:**
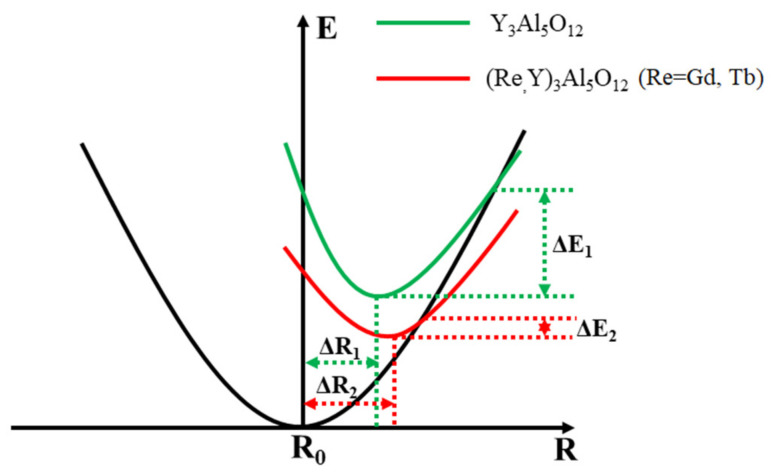
Configuration coordinate diagrams of Y_3_Al_5_O_12_ and (Re,Y)_3_Al_5_O_12_.

This phenomenon could be attributed to the ionic radius of Gd^3+^/Tb^3+^ being larger than that of the Y^3+^ ion, resulting in a reduced lattice rigidity of the garnet structure. Specifically, the configurational coordination diagram of Ce^3+^ can be used to qualitatively elaborate the thermal quenching mechanism of Ce/Mn/Cr: (Re_x_Y_1−x_)_3_Al_5_O_12_ TCs, as shown in Figure 6, with ΔR being the difference between the equilibrium locations of Ce^3+^ ion’s ground-state and excited-state potential curves. The elevated lattice expansion of the garnet structure caused a substantial distortion in [Gd^3+^/Tb^3+^O_8_], resulting in an augmented ΔR, a reduced rigidity and activation energy ΔE, and ultimately leading to the observed degradation in thermal stability. Based on empirical evidence, it is feasible to fit the activation energy ΔE using the Arrhenius equation:

(3) $$I\left(T\right)=\frac{I_{0}}{1+A\mathrm{e}\frac{-\Delta E}{kT}}$$

where *I*_0_ is the initial emission intensity at room temperature, *I*(*T*) is the emission intensity at operating temperature, *A* is a constant, *k* is the Boltzmann constant (8.617 × 10^−5^ eV·K^−1^) and Δ*E* is the activation energy of thermal quenching [48,49]. Through the utilization of Equation (3), the relationship of Ln (I_0_/I_T_ − 1) versus 1/T can be plotted as seen in Figure 5h, where the inclination of the resulting line represents the activation energy, as denoted by ΔE. The calculated ΔE of Ce/Mn/Cr: (Re_x_Y_1−x_)_3_Al_5_O_12_ TCs for S1, S2, S3, S5, S6, S7 and S8 are 0.442, 0.361, 0.409, 0.345, 0.391, 0.338 and 0.302 ev, respectively. The substitution of Gd and Tb ions generally results in an increased displacement of ionic equilibrium positions, leading to a rightward and upward shift in the excited state curve in the configuration coordinate diagrams and a decrease in activation energy. Conversely, the effect caused by Lu ions is the opposite. But in our test results, the S8 sample also exhibited a reduced activation energy. This could be attributed to the fact that the sintering temperature of the S8 did not reach its optimum, leading to a higher concentration of internal defects within it. The thermal stability may decrease due to defects and impurities, which would become the non-radiative transition center and lowering its thermal stability. In this study, in order to maintain consistency in sample preparation, we did not optimize the sintering condition for all the samples, and the optical quality of the samples is not uniform. Furthermore, by altering the composition of the base materials by introducing Gd, Tb and Lu, the bandgap as well as the electron thermal ionization from the 5d to the conduction band would also be changed, which will also affect the thermal stability of the material.

**Figure 7:**
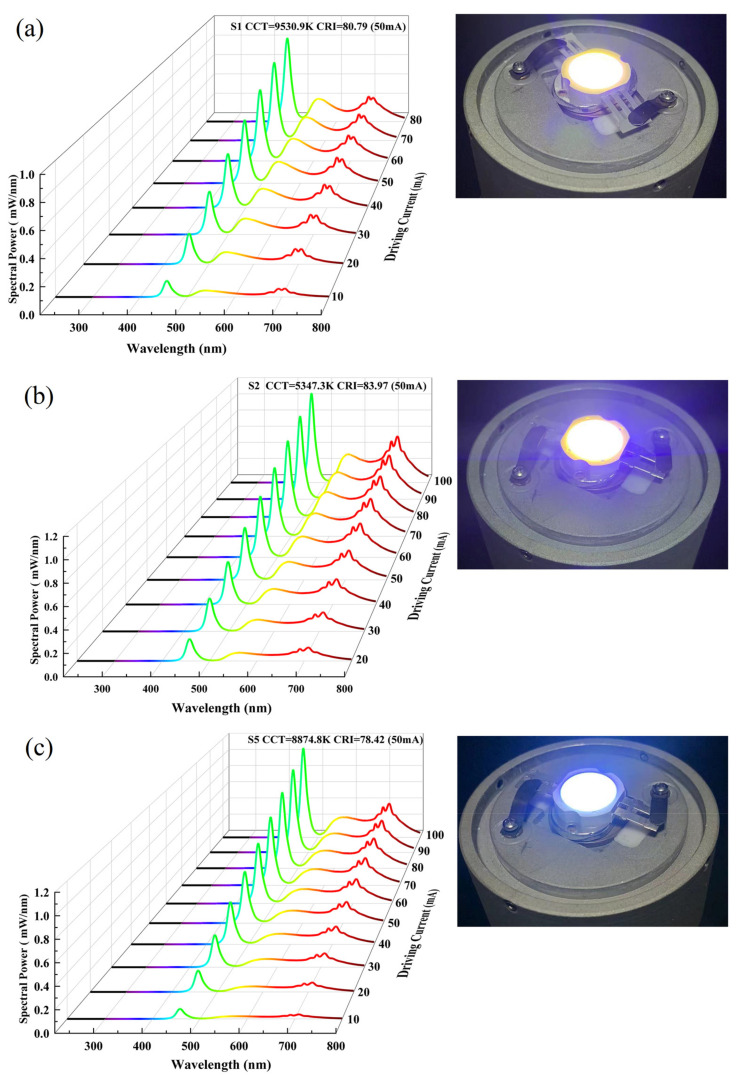
Electroluminescence spectra of the WLED composed of S1, S2 and S5 TCs under different current excitation (**a**–**c**); the insets are the real experimental scene of the TCs mounted to the 450 nm blue LED chips.

Given that the predominant emission mode in current practical applications of LEDs is transmissive, we employed S1, S2 and S5 samples with higher transmittance to mount onto commercial blue LED chips for the measurement of obtained white light colorimetric parameters. As shown in Figure 7, with the increasing driving current, the EL intensity of WLEDs increased, and no luminescence saturation was observed before 100 mA. Under a driving current of 50 mA, the white-light LED devices encapsulated with S1, S2 and S5 samples exhibited a CRI of 80.79, 83.97 and 78.42, respectively, which was significantly higher than that of white-light LEDs encapsulated with conventional Ce: YAG ceramics or phosphors. However, it should be noted that the intense energy transfer from Ce^3+^ to Cr^3+^ and Mn^2+^ may lead to a significant reduction in Ce^3+^ luminescence when the red part of the spectral distribution is enhanced, resulting in further adjustments to the CRI and a decrease in the luminous flux.

## 4. Conclusions

The Ce/Mn/Cr: (Re,Y)_3_Al_5_O_12_ (Re = Gd, Tb and Lu) phosphor ceramics were fabricated via solid-state reaction and vacuum sintering. By doping Cr^3+^ and Mn^2+^, the red component of Ce^3+^ spectra can be effectively enhanced. The Ce^3+^ can act as both a sensitizer and an activator, and the intense energy transfer from Ce^3+^ ions to Mn^2+^/Cr^3+^ ions has been realized. By introducing Gd and Tb into YAG lattice, the formed [GdO_8_] and [TbO_8_]’s crystal field splitting was more significant than that of [YO_8_], resulting in the massive redshift of Ce^3+^ emission spectra, whereas [LuO_8_] has the opposite effect, causing the blueshift of the emission peak. The white-light LED devices encapsulated with Ce/Mn/Cr: (Gd_0.333_Y_0.664_)_3_Al_5_O_12_ exhibited a high CRI of 83.97. The study provides insights into improving the Color-Rendering Index of white LED lighting. In our future research plans, we intend to explore Spark Plasma Sintering (SPS) and Hot Isostatic Pressing (HIP) sintering techniques for improved quality of target phosphor ceramics. During the sintering process, we also aim to further optimize the heating and cooling rates [50] as well as the sintering soaking time. Additionally, the degradation of thermal stability should be alleviated by designing a more symmetrical structure with a higher stiffness.

## Figures and Tables

**Table 1:** Ingredients of the (Y_0.997−x_Re_x_Ce_0.003_)_3_(Al_0.9748_ Mn^2+^_0.024_Cr^3+^_0.0012_)_5_O_12_ TCs.

Sample No	Stoichiometry	Chemical Formula
S1	Re_x_ = 0	(Y_0.997_Ce_0.003_)_3_(Al_0.9748_ Mn^2+^_0.024_Cr^3+^_0.0012_)_5_O_12_
S2	Re_x_ = Gd_0.333_	(Y_0.664_Gd_0.333_Ce_0.003_)_3_(Al_0.9748_ Mn^2+^_0.024_Cr^3+^_0.0012_)_5_O_12_
S3	Re_x_ = Gd_0.666_	(Y_0.331_Gd_0.666_Ce_0.003_)_3_(Al_0.9748_ Mn^2+^_0.024_Cr^3+^_0.0012_)_5_O_12_
S4	Re_x_ = Gd_0.997_	(Gd_0.997_Ce_0.003_)_3_(Al_0.9748_ Mn^2+^_0.024_Cr^3+^_0.0012_)_5_O_12_
S5	Re_x_ = Tb_0.333_	(Y_0.664_Tb_0.333_Ce_0.003_)_3_(Al_0.9748_ Mn^2+^_0.024_Cr^3+^_0.0012_)_5_O_12_
S6	Re_x_ = Tb_0.666_	(Y_0.331_Tb_0.666_Ce_0.003_)_3_(Al_0.9748_ Mn^2+^_0.024_Cr^3+^_0.0012_)_5_O_12_
S7	Re_x_ = Tb_0.997_	(Tb_0.997_Ce_0.003_)_3_(Al_0.9748_ Mn^2+^_0.024_Cr^3+^_0.0012_)_5_O_12_
S8	Re_x_ = Lu_0.997_	(Lu_0.997_Ce_0.003_)_3_(Al_0.9748_ Mn^2+^_0.024_Cr^3+^_0.0012_)_5_O_12_

## Data Availability

The data presented in this study are available on request from the corresponding author. The data are not publicly available due to relevant research is still in progress.
